# A Targeted Library Screen Reveals a New Inhibitor Scaffold for Protein Kinase D

**DOI:** 10.1371/journal.pone.0044653

**Published:** 2012-09-18

**Authors:** Manuj Tandon, Lirong Wang, Qi Xu, Xiangqun Xie, Peter Wipf, Qiming Jane Wang

**Affiliations:** 1 Department of Pharmacology and Chemical Biology, University of Pittsburgh, Pittsburgh, Pennsylvania, United States of America; 2 Department of Chemistry and Center for Chemical Methodologies and Library Development, University of Pittsburgh, Pittsburgh, Pennsylvania, United States of America; 3 Department of Pharmaceutical Sciences, University of Pittsburgh, Pittsburgh, Pennsylavania, United States of America; Albert-Ludwigs-University, Germany

## Abstract

Protein kinase D (PKD) has emerged as a potential therapeutic target in multiple pathological conditions, including cancer and heart diseases. Potent and selective small molecule inhibitors of PKD are valuable for dissecting PKD-mediated cellular signaling pathways and for therapeutic application. In this study, we evaluated a targeted library of 235 small organic kinase inhibitors for PKD1 inhibitory activity at a single concentration. Twenty-eight PKD inhibitory chemotypes were identified and six exhibited excellent PKD1 selectivity. Five of the six lead structures share a common scaffold, with compound **139** being the most potent and selective for PKD vs PKC and CAMK. Compound **139** was an ATP-competitive PKD1 inhibitor with a low double-digit nanomolar potency and was also cell-active. Kinase profiling analysis identified this class of small molecules as pan-PKD inhibitors, confirmed their selectivity again PKC and CAMK, and demonstrated an overall favorable selectivity profile that could be further enhanced through structural modification. Furthermore, using a PKD homology model based on similar protein kinase structures, docking modes for compound **139** were explored and compared to literature examples of PKD inhibition. Modeling of these compounds at the ATP-binding site of PKD was used to rationalize its high potency and provide the foundation for future further optimization. Accordingly, using biochemical screening of a small number of privileged scaffolds and computational modeling, we have identified a new core structure for highly potent PKD inhibition with promising selectivity against closely related kinases. These lead structures represent an excellent starting point for the further optimization and the design of selective and therapeutically effective small molecule inhibitors of PKD.

## Introduction

Protein kinase Ds (PKDs) are diacylglycerol (DAG)-regulated serine/threonine protein kinases that belong to a distinct subgroup of the calcium/calmodulin-dependent protein kinase (CAMK) family [1], [2]. The binding of DAG occurs at a conserved C1 domain shared among DAG receptors including the protein kinase C (PKC) family. Structurally, the catalytic domain of PKD bears a high resemblance to those of CAMKs. In intact cells, PKD is activated by DAG-responsive PKCs through phosphorylation of two conserved serine residues in the activation loop of the catalytic domain. The DAG/PKC/PKD axis is recognized as a major signaling pathway for the regulation of a variety of important biological events [3].

The three isoforms of PKD (PKD1, PKD2 and PKD3) have emerged as key mediators in cellular processes pertaining to multiple diseases, including cancer, heart diseases, angiogenesis-related diseases and immune dysfunctions [3], [4]. In particular, PKD has been implicated in many aspects of tumor development, such as tumor growth, metastasis, and angiogenesis [4]. Aberrant PKD activity and expression have been reported in various tumor cell lines and tumor tissues from the pancreas [5], skin [6], [7] and prostate [8], [9]. PKD has been shown to mediate major signaling pathways that are vital to cancer development, including the VEGF and MEK/ERK signaling pathways [4], thus supporting an active role of PKD in tumor-associated biological processes in diverse cancer types [5], [7], [9], [10], [11], [12]. PKD is a viable target in hypertrophic response of the heart by acting on its substrates, the class IIa histone deacetylases (HDAC 4, 5, 7, 9). Of particular note is the role of PKD in cardiac hypertrophy where it regulates HDAC5 [13], [14], [15]. Previous studies have identified PKD phosphorylation and induction of nuclear exclusion of HDAC5 as a mediator of persistent stress-induced cardiac hypertrophy [15]. Ectopic overexpression of constitutively active PKD1 in mouse heart leads to cardiac hypertrophy [14], [15], [16], while cardiac-specific deletion of PKD1 in mice suppressed pathological cardiac remodeling in response to various stress stimuli and significantly improved cardiac function [13], indicating a critical role of PKD in this pathological process. Taken together, PKD has emerged as a potential therapeutic target for cancer, cardiac hypertrophy, and other diseases.

With the growing evidence supporting an important role of PKD in various pathological conditions, the discovery and development of potent and selective PKD modulators have accelerated in recent years. In addition to the pan-kinase inhibitors staurosporine and K252a (25), a number of novel, potent and structurally distinct PKD inhibitors have been reported. These include CID755673 and analogs [17], [18], 2,6-naphthyridine and bipyridyl inhibitors and their analogs [19], [20], [21], 3,5-diarylazoles [22], CRT0066101 [23], and CRT5 [24], all showing nanomolar inhibitory activities towards PKD. In general, these inhibitors are equally potent for all PKD isoforms, and none of them have progressed to the clinic, most likely due to lack of selectivity, *in vivo* stability and general toxicity issues. Accordingly, the search for novel PKD inhibitory chemotypes with appropriate selectivity profiles and high *in vivo* efficacy continues unabated. An ideal inhibitor would not only provide more opportunities for the translation of PKD inhibitors to the clinic, but also provide a useful tool for dissecting PKD-mediated signaling pathways and biological processes in cellular and *in vivo* settings.

**Figure 1:**
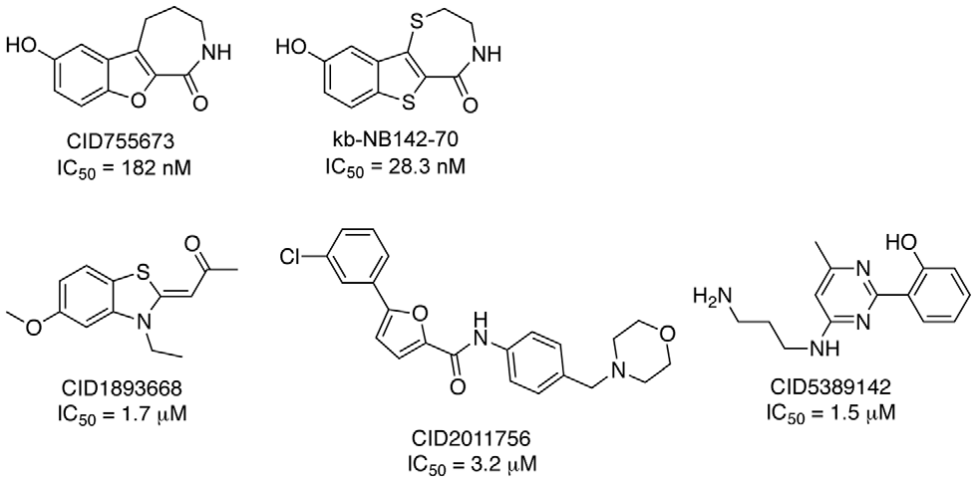
Structures and PKD1 inhibitory activities of selected small molecule inhibitors identified in previous HTS assays.

In previous work, we took advantage of HTS campaigns of large, unbiased small molecule libraries to identify novel inhibitors, and applied medicinal chemistry strategies to optimize activity, selectivity, and physicochemical properties [17], [18], [25], [26], [27]. This approach provided both ATP-competitive active site, and non-competitive, presumably allosteric site inhibitors (**Fig. 1**). For example, CID755673 and kb-NB142-70 inhibited PKD1 *in vitro* in the low nanomolar range and suppressed PKD1 autophosphorylation at Ser^916^ in LNCaP prostate cancer cells in the low micromolar range. CID1893668, CID2011756, and CID5389142 also inhibited phorbol ester-induced endogenous PKD1 activation in LNCaP prostate cancer cells in a concentration-dependent manner.

Using a small, targeted library of diverse kinase inhibitors, we have now identified twenty-eight new ATP-competitive inhibitors of PKD. Among these, eight displayed excellent selectivity towards PKD with little or no inhibitory activity for CAMK or PKC, two structurally and functionally closely related kinases. Additionally, we have developed a homology model of PKD and investigated at the molecular level the interactions of these PKD inhibitors in the active site of the kinase. The newly discovered PKD inhibitors hold promise for the further development of clinically effective PKD-specific inhibitors.

## Results

### Screening of a kinase inhibitor library reveals novel PKD1 inhibitory chemotypes

**Figure 2:**
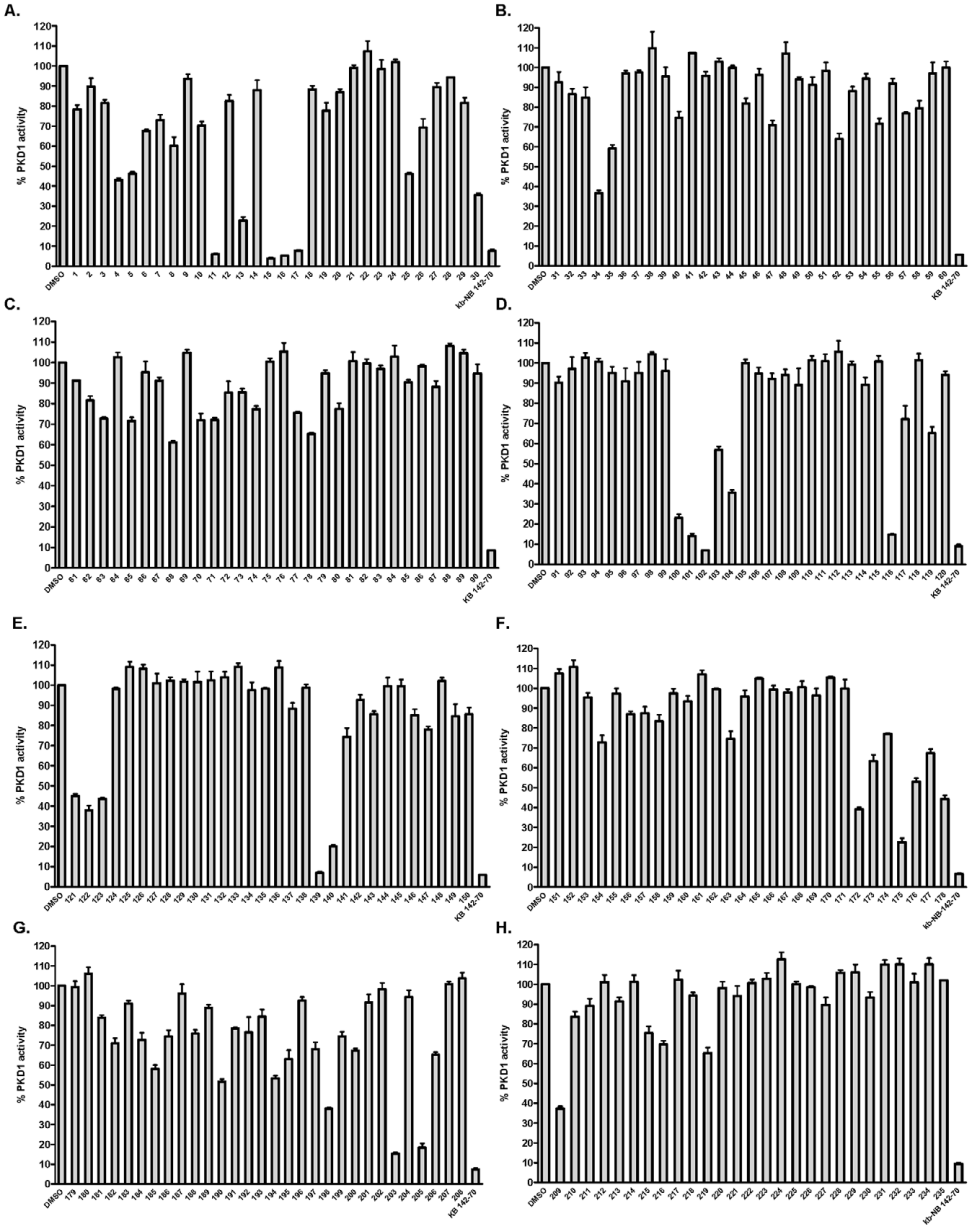
Screen of a kinase inhibitor library for PKD1 activity. A targeted library of 235 compounds was screened for PKD1 activity at 1 µM using an *in vitro* radiometric PKD1 kinase assay. The representative graphs show % residual PKD1 kinase activity calculated based on the total kinase activity measured in the absence of inhibitors (DMSO). Kb-NB142-70, a previously known PKD inhibitor, was used as a positive control. Experiments were performed with triplicate determinations at 1 µM for each compound.

**Figure 3:**
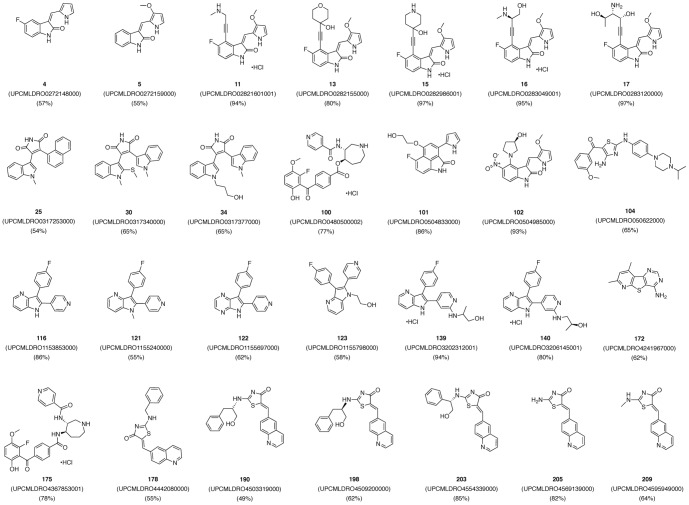
Chemical structures of novel PKD1 small molecule inhibitors identified from the screen. Twenty-eight PKD1 inhibitors were identified as primary hits in a screen using a radiometric PKD1 kinase assay. Hits were selected based on their ability to inhibit PKD1 at or above 50% at 1 µM.

A collection of 235 unique small molecule kinase inhibitors was obtained from Hoffmann-La Roche, Inc. To search for active site-targeted novel inhibitory chemotypes of PKD, an *in vitro* screen was conducted on PKD1 using an established radiometric PKD1 kinase assay [17]. All compounds were given distinct UPCMLD (University of Pittsburgh Center for Chemical Methodologies and Library Development) IDs and were then assigned numerical IDs for convenient data display. Evaluation of each compound was carried out in triplicate at a single concentration (1 µM). Our previously described PKD1 inhibitor, kb-NB142-70, and DMSO (solvent alone) were used as positive and negative controls, respectively. Percent residual kinase activity was calculated based on that of the negative control (DMSO). Compounds with 50% or greater inhibitory activity for PKD1 were selected as primary hits (**Fig. 2A–H**). A total of twenty-eight kinase inhibitors were thus identified as PKD inhibitors in this screen (a hit ratio of 12%) (**Fig. 3**).

### Identification of PKD1 inhibitors with desired selectivity profile

The specificity of the newly identified PKD1 inhibitors was assessed using *in vitro* kinase assays against PKC and CAMK, two families of kinases functionally and structurally related to PKD. PKC, like PKD, is a DAG/phorbol ester receptor and a direct activator of PKD. The PKC/PKD pathway is a key signaling pathway that accounts for PKD-mediated cellular responses [28], [29]. The kinase domain of PKD bears high sequence homology to the CAMK family of kinases. Functionally, CAMK also partially overlaps with PKD in regulation of certain substrates and signaling events; for example, both kinases phosphorylate class IIa HDACs and have been implicated in cardiac hypertrophy. Thus, selectivity against these two related kinase families is a highly desirable feature of a specific PKD inhibitor.

**Figure 4:**
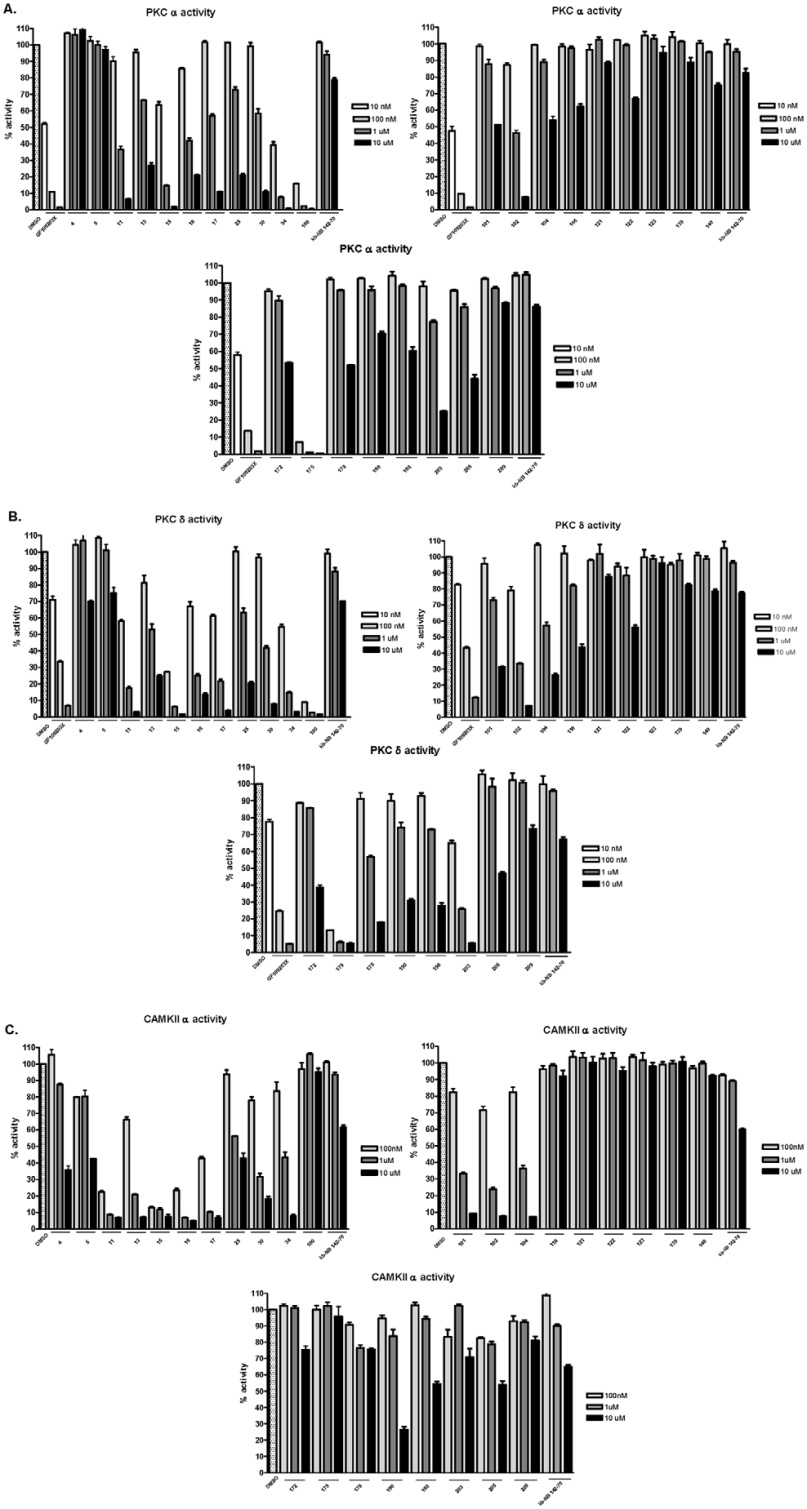
Selectivity of twenty-eight PKD1 inhibitors. Inhibition of PKCα (**A**), PKCδ (**B**), or CAMKIIα (**C**) by each of the twenty-eight hits was determined at 100 nM, 1 µM and 10 µM concentrations. In the PKC assays, GF109203X, a potent PKC inhibitor was used as control.

In this study, we counter-screened the twenty-eight PKD1 inhibitory agents for inhibition of PKCα, PKCδ and CAMKIIα in order to get an initial profile for the potential PKD selectivity, since these are the functionally most closely related kinases. The compounds were examined at 0.1, 1 and 10 µM concentration. Inhibitors that exhibited ≤50% inhibition at the highest concentration (10 µM) were considered “inactive” for PKC or CAMK. As shown in **Fig. 4A** **and** **Table 1**, fifteen compounds were identified as “inactive” inhibitors of PKCα. Compounds **116,** **190**, and **198** showed nearly 70% inhibition of PKCδ at 10 µM, and **101**, **104**, **172**, and **178** were near the 50% cut-off value. The remaining eight compounds fit our criteria of “inactive” inhibitors of PKCδ (**Fig. 4B**, **Table 1**). When used as a positive control, the potent PKC inhibitor GF109203X strongly inhibited both PKC isoforms in concentration-dependent manner. Next, the inhibitory activity for CAMKIIα was examined. As shown in **Fig. 4C** and **Table1**, a total of fourteen compounds were found to be “inactive” for CAMKIIα. Overall, among the twenty-eight hits, we identified twelve PKD1 inhibitors that lacked activity or were poorly inhibitory for at least two of out of the three undesired kinase targets (PKCα, PKCδ and CAMKIIα. Among them, six (**121**, **122**, **123**, 139, **140**, **209**) were considered “inactive” for all three kinases, suggesting excellent selectivity for PKD1 relative to these two families of kinases.

**Table 1:** PKD1 selective inhibitors with little or no inhibitory activity for PKCα, PKCδ or CAMKIIα.

Inactive compds for PKCα	Inactive compds for PKCδ	Inactive compds for CAMKIIα
4	4	-
5	5	-
101*	-	-
104*	-	-
116	-	116
**121**	**121**	**121**
**122**	122*	**122**
**123**	**123**	**123**
**139**	**139**	**139**
**140**	**140**	**140**
172*	-	172
178*	-	178
190	-	-
198	-	198*
-	-	203
-	-	205*
**209**	**209**	**209**
-	-	100
-	-	175

A list of PKD inhibitors that had ≤50% inhibitory activity for PKCα, PKCδ or CAMKIIα at 10 µM. Compounds 121, 122, 123, 139, 140, 209 (bold) were identified as “inactive” compounds for all three kinases.

### *In vitro* IC_50_, cellular activity, and mode of action of novel PKD1 inhibitor scaffolds

A structural analysis of the six most selective PKD1 inhibitors revealed two distinct scaffolds. One chemotype is represented by 3-(4-fluorophenyl)-2-(pyridin-4-yl)-1*H*-pyrrolo[3,2-b]pyridine, also known as 4- or 4,7-azaindole. A total of seven 4-azaindoles were part of the library. Six were identified as PKD1 inhibitors in the screen and five (i.e. **121**, **122**, **123**, **139** and **140**) exhibited exclusive inhibitory activity for PKD1. Thus, this scaffold appears to be highly specific for PKD1. Moreover, the PKD1 inhibitory activity of the five analogs varied widely from borderline (55%) to the highest (94%) inhibition of total PKD1 activity, suggesting that the scaffold is readily amenable to chemical optimization for enhanced specificity. A second chemotype (a quinolinyl-methylenethiazolinone), a singleton, was represented by compound **209**. A structural analysis of the library revealed a total of thirty-four quinolinyl-methylenethiazolinones, and five of these were identified as PKD1 inhibitors. However, only one member of this chemotype inhibited PKD1 exclusively, with the rest being active against at least one of the undesired PKC or CAMK kinase targets. Thus, this scaffold appears to be more promiscuous and less promising in comparison to the 4-azaindoles.

To further evaluate the chemical structures of these hits, we employed a computational approach to evaluate the structural similarity of these compounds to known PKD inhibitors. As shown in **Table S1**, our data derived from both 2D Tanimoto score (TS) and 3D morphological similarity score (MSS) analyses indicate that the six novel PKD1 inhibitors display weak (MSS, 3–5; TS, 0.1–0.3) structural similarity to most of the known PKD1 inhibitors, with a few exhibiting moderate (MSS = 6–7 and TS = ∼0.4) similarity, supporting the novelty of these structures as PKD1 inhibitors.

**Figure 5:**
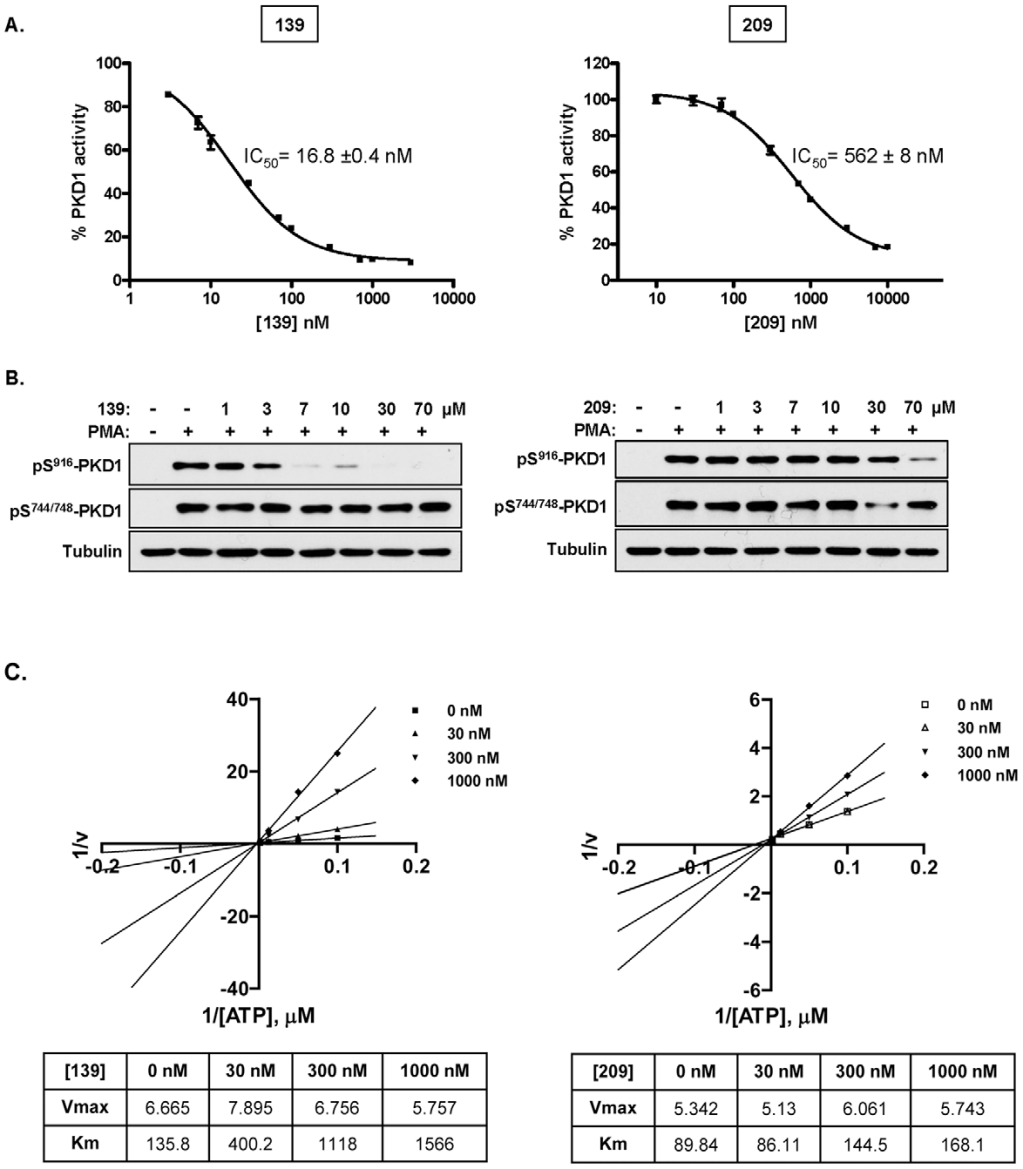
*In vitro* IC_50_, cellular activity, and mode of action of representative compounds. **A**. Concentration-dependent inhibition of PKD1 *in vitro* by two representative compounds, **139** and **209**. IC_50_ values were calculated based on the dose-response curve. Data are represented as mean ± SEM of 3 independent experiments. **B**. Inhibition of endogenous PKD1 activity by the compounds in intact cells. LNCaP prostate cancer cells were pretreated with increasing concentration of compounds **139** and **209** for 45 min, followed by PMA stimulation at 10 nM for 20 min. Cell lysates were subjected to immunoblotting for pS^916^-PKD1 and pS^744/748^-PKD1. Tubulin was blotted as loading control. The experiments were repeated three times and representative blots are shown. **C**. Both compounds are ATP-competitive inhibitors. Lineweaver-Burk plots of compounds **139** and **209**. V_max_ and K_m_ values derived from the plots are shown in the table below each plot. Data are representative of three independent experiments.

Next, a representative example for each scaffold, compounds **139** and **209**, was evaluated in secondary assays for *in vitro* and cellular activities and mode of action. As shown in **Fig. 5A**, compound **139** inhibited PKD1 *in vitro* in a concentration dependent manner with an IC_50_ of 16.8 nM, while compound **209** inhibited with an IC_50_ = 562 nM. To determine if the compounds were active PKD1 inhibitors in cells, we determined their ability to inhibit phorbol 12-myristate 13-acetate (PMA)-induced activation of PKD1 in LNCaP prostate cancer cells. PMA induces PKC-dependent phosphorylation of Ser744/748 (S^744/748^) in the activation loop followed by autophosphorylation of PKD1 on Ser916 (S^916^) in the C-terminus [30], [31]. The catalytic activity of PKD1 correlates well with the level of phosphorylation at S^916^ [31]. As illustrated in **Fig. 5B**, compounds **139** and **209** blocked PMA-induced autophosphorylation at S^916^ in a concentration dependent fashion, but did not affect PKC-induced transphosphorylation at S^744/748^. This result is consistent with the notion that both inhibitors directly target PKD1 and do not interfere with the activity of upstream PKCs. The cellular IC_50_s for inhibition of PKD1 obtained from the densitometry analysis of pS^916^-PKD1 levels were 1.5 µM for compound **139** and 18.2 µM for compound **209**, in good correlation with their *in vitro* activities for PKD1. Finally, kinetic analyses confirmed that both compounds were competitive with respect to ATP (**Fig. 5C**). Taken together, compounds **139** and **209** are potent (compound **139**) and cell-active ATP-competitive PKD1 inhibitors.

### Selectivity profile of 4-azaindoles

**Figure 6:**
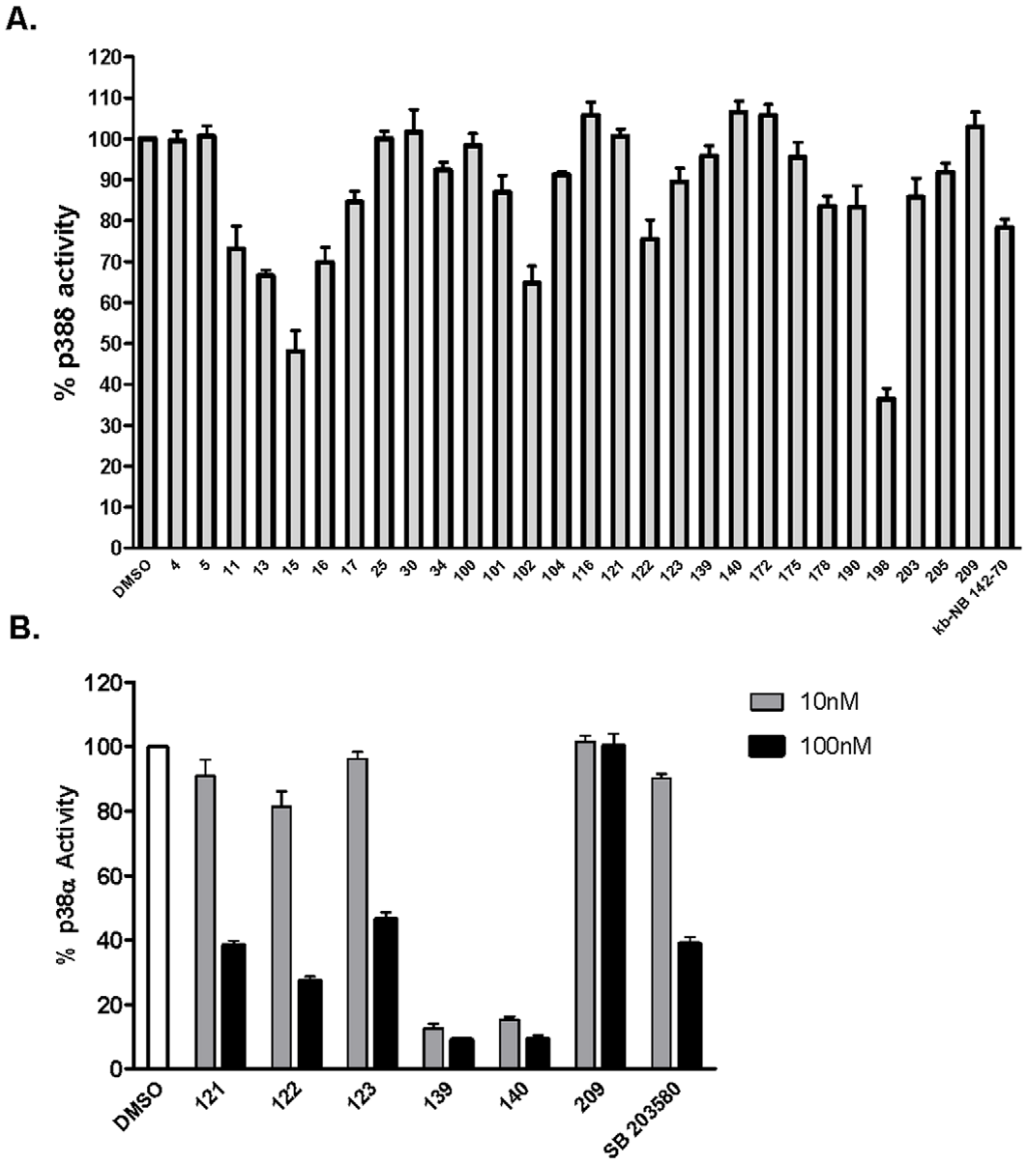
Inhibition of p38δ and p38α by the PKD1 inhibitors. Inhibitory activities of the twenty-eight hits for p38δ at 1 µM (**A**) and the six most selective inhibitors for p38α at 10 and 100 nM (**B**) were evaluated using an *in vitro* p38 kinase assay. The representative graphs show % residual p38 kinase activity calculated based on the total kinase activity measured in the absence of inhibitors (DMSO). The experiment was performed twice with triplicate determinations at 1 µM for each compound and a representative graph is shown.

To assess if additional targets existed for the two novel PKD1 inhibitor scaffolds, extensive data mining of similar structures was conducted and revealed one additional target for each scaffold [32], [33]. The 4-, or 4,7-azaindoles have been reported as potent inhibitors of p38α MAP kinase [32], while quinolinylmethylenethiazolinones were found to inhibit CDK1/cyclin B [33]. Since quinolinylmethylenethiazolinones proved promiscuous based on our previous analysis, our primary focus was on the 4-azaindoles. To confirm that p38 was indeed a target of the 4-azaindole, the inhibition of p38 by the twenty-eight PKD1 inhibitors was evaluated using an *in vitro* radiometric p38 kinase assay. Interestingly, as shown in **Fig. 6A**, with the exception of compounds **15** and **198**, none of the twenty-eight hits exhibited ≥50% inhibition of p38δ at 1 µM, and the six most selective compounds for PKD1 in particular showed none or less than 20% inhibition of p38δ at 1 µM. In contrast, when a different isoform – p38α – was evaluated, the six lead compounds, with the exception of compound **209**, significantly inhibited p38α in a concentration-dependent manner at 10 and 100 nM, with compounds **139** and **140** being most potent (**Fig. 6B**). When used as a control, the p38 inhibitor SB 203580 resulted in >60% inhibition of p38α at 100 nM. These results are consistent with the previous report, indicating that 4- or 4,7-azaindoles were selective inhibitors of p38α, but not p38δ [32]. Taken together, our data has identified a novel PKD1 inhibitor scaffold that is active for p38α, but not for PKC, CAMK and p38δ.

**Figure 7:**
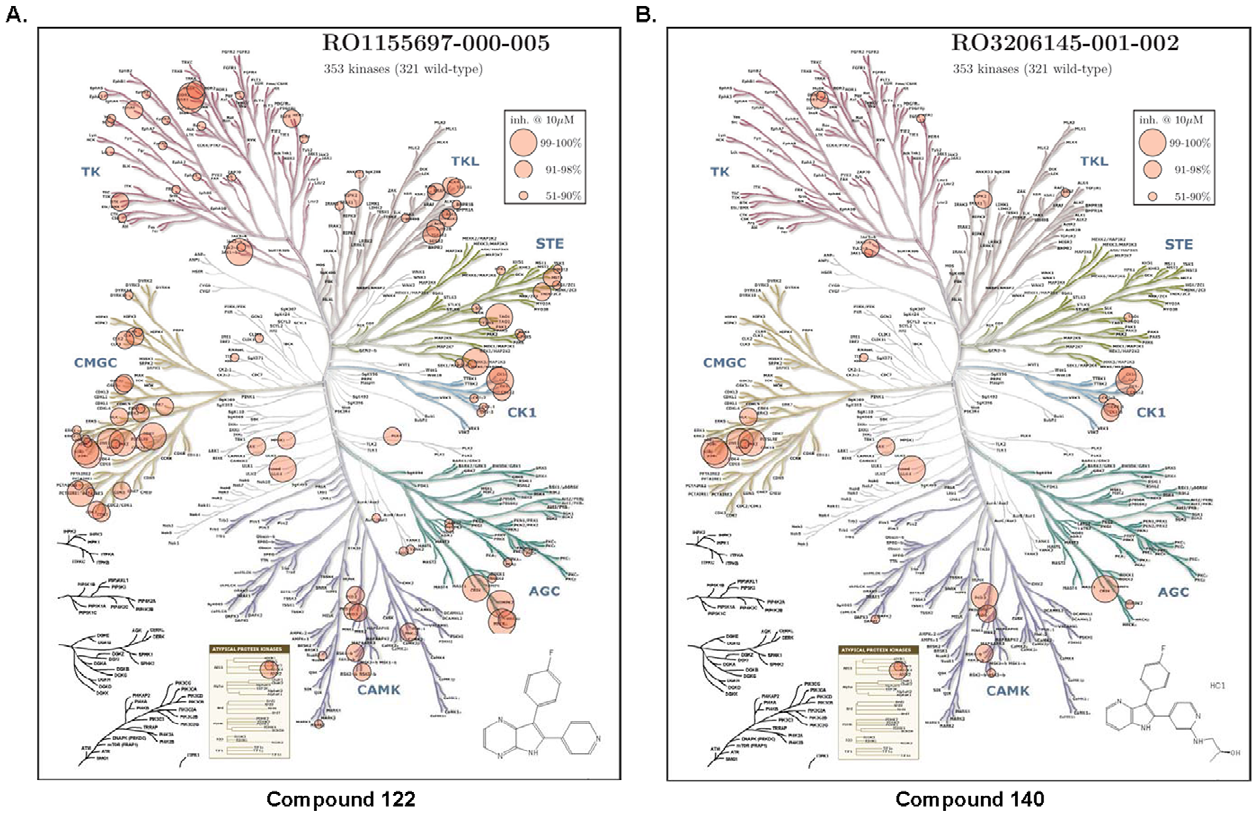
The selectivity of compounds 122 and 140 presented on a dendrogram of the human kinome. **A**. Compound 122 at 10 µM. **B**. Compound 140 at 10 µM. Pink circle represents inhibitory activity: *big circle*, 99–100% inhibition; *intermediate circle*, 91–98% inhibition; *small circle*, 51–90% inhibition.

To obtain a more complete selectivity profile for the 4-azaindole scaffold, a global kinase profile of compounds **122** and **140** was conducted at 10 µM on 353 kinases representing seven groups of eukaryotic protein kinases (ePKs) and one group of atypical protein kinases (aPKs) (**File S1**). This profiling approach uses an active site-directed competition binding assay to quantify the interactions of test compounds and kinases. Compound **122** is a 4,7-azaindole that has no substitutions on the indole ring. Compound **140**, similar to **139**, possesses a hydroxyalkylamine substitution (2-hydroxypropyl for **140** and 2-hydroxyl-1-methylethyl for **139**) on the pyridyl ring at the *ortho*-position to the nitrogen, and exhibited similar biological activities as **139** [32]. As shown in **Table 2**, the binding activity of 123 out of the 353 kinases was inhibited at over 50% by compound **122**, representing 35% of the total kinases tested, while a significantly smaller number of kinases (43, 12%) was inhibited at this level by compound **140**. Correspondingly, the number of protein kinases in the three competition levels (99–100%, 91–98%, 51–90%) was dropped from 17, 47, and 59 for compound **122** to 8, 12, and 23 for compound **140**, respectively, indicating a significant improvement in selectivity. This is also evident from the compound interactions mapped across the human kinase dendrogram (**Fig. 7**). Profiling of compound **140** revealed a total of eight protein kinases that were inhibited by compound **140** at the 99–100% level, including five kinase families, PRKD (PKD2), p38 (p38α, β), JNK (JNK1, 2), STK (STK36, CIT/STK21), and CSNKIE (CK1ε) (**Table 2** **and File S1**). For the PKD family, all three isoforms were inhibited by **140** with similar potencies (PKD1, 83%; PKD2, 99%; PKD3, 96%), as found for **122**, which agrees with our previous results and indicates that 4-azaindoles are pan-PKD inhibitors. Also included in the kinase profile are four isoforms of PKC (δ, ε, η, θ) and eight isoforms of CAMKs (CAMKIα, δ, γ; CAMKIIα, β, δ, γ; CAMK4). With the exception of PKC θ that was weakly inhibited by compound **122** (78% at 10 µM), none was affected (<50% competition) by compounds **122** or **140**, further supporting our conclusion that 4-azaindoles are preferred PKD inhibitors with selectivity against PKCs and CAMKs.

**Table 2:** Selectivity profiling of compounds 122 and 140.

Ambit Gene Symbol	% Competition		Ambit Gene Symbol	% Competition	
	*122*	*140*		*122*	*140*
ACVR1	92	<50	**JNK1**	100	**100**
ACVR1B	91	<50	**JNK2**	100	**100**
ACVR2B	92	<50	JNK3	88	83
ACVRL1	83	<50	KIT(V559D)	75	<50
ADCK3	83	90	LATS1	68	<50
ADCK4	94	98	LATS2	82	<50
AURKC	86	<50	LCK	71	<50
BMPR1B	81	<50	MAP4K4	84	<50
BRAF	97	76	MARK2	77	<50
BRAF(V600E)	97	78	MEK3	99	<50
BTK	91	<50	MEK4	87	<50
CDC2L2	92	<50	MEK6	66	<50
CDK2	93	<50	MRCKA	99	<50
CDK3	92	<50	MRCKB	98	<50
CDK5	92	<50	MST3	68	<50
CDK7	100	<50	MST4	97	<50
**CIT**	99	**99**	MUSK	94	<50
CLK1	97	<50	NLK	98	91
CLK2	97	<50	**p38-alpha**	100	**100**
CLK4	88	<50	**p38-beta**	100	**100**
CSK	71	<50	p38-delta	82	<50
CSNK1A1L	95	86	p38-gamma	90	<50
CSNK1D	97	96	PAK4	82	<50
**CSNK1E**	100	**100**	PAK7	86	<50
CSNK1G1	78	94	PCTK1	100	<50
CSNK1G2	82	89	PCTK2	78	<50
CSNK1G3	86	91	PCTK3	77	<50
DAPK1	<50	73	PHKG2	91	<50
DDR1	100	98	PKAC-beta	67	<50
DDR2	100	67	PLK4	98	<50
DMPK2	100	74	PRKCQ	78	<50
DYRK1B	79	<50	PRKD1	92	83
EGFR	95	76	**PRKD2**	98	**99**
EGFR(E746-A750del)	96	90	PRKD3	98	96
EGFR(G719C)	99	87	RAF1	81	<50
EGFR(G719S)	96	70	RET	68	<50
EGFR(L747-E749del, A750P)	98	90	RET(M918T)	68	<50
EGFR(L747-S752del, P753S)	95	84	RIPK2	97	94
EGFR(L747-T751del,Sins)	97	90	RIPK4	76	<50
EGFR(L858R)	98	84	ROCK2	89	<50
EGFR(L861Q)	97	87	ROS1	80	<50
EGFR(S752-I759del)	98	91	RPS6KA1(Kin.Dom.2-C-terminal)	92	87
EPHA1	81	<50	RPS6KA6(Kin.Dom.2-C-terminal)	98	96
EPHA3	85	<50	SLK	80	<50
EPHA6	93	<50	SNARK	89	<50
EPHA8	71	<50	SRC	87	<50
EPHB3	82	<50	STK16	96	<50
EPHB4	72	<50	STK35	79	<50
ERBB4	80	<50	**STK36**	100	**100**
ERK1	80	<50	SYK	72	<50
ERK2	87	<50	TAOK1	99	67
ERK3	98	<50	TAOK3	80	<50
ERK4	68	<50	TGFBR1	91	<50
ERK8	98	<50	TGFBR2	98	<50
FLT3(D835Y)	76	<50	TNIK	96	<50
FRK	85	<50	TNNI3K	67	<50
GAK	98	98	TTK	88	90
GSK3A	98	<50	TYK2 (JH2 domain-pseudokinase)	78	92
GSK3B	96	<50	YANK2	89	<50
HPK1	67	<50	YANK3	67	<50
IRAK3	75	<50	YES	73	<50
JAK1 (JH2 domain-pseudokinase)	99	90			

Listed in this table are all the protein kinases that bound compound **122** at over 50% at 10 µM. Their competition by compound **140** is listed in parallel. Compound **140** exhibited greater selectivity as compared to compound **122**. The data were obtained from profiling of a total of 353 kinases in the kinome. Enzymes competed by compound **140** at 99–100% are bolded.

### Structure modeling of PKD1 kinase domain

To further explore the mechanism of action of these active PKD1 compounds, molecular modeling technologies were utilized to investigate the putative binding modes using our reported protocols [34], [35]. The three-dimensional structure of PKD1 and the catalytic (kinase) domain which consists of two lobes and an intervening linker was built based on high-resolution crystal structures of homologues.

**Figure 8:**
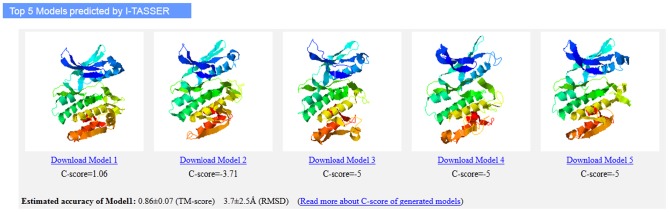
Five models of human PKD1 kinase domain generated by I-TASSER server.

The sequence of the PKD1 kinase domain, which extends from Glu587 to Ser835, was submitted to the I-TASSER server for 3D structure prediction. Protein structures 1ql6_A (rabbit, phosphorylase kinase), 2bdw_A (caenorhabditis elegans, calcium/calmodulin activated kinase II), 3mfr_A (human, calcium/calmodulin (CaM)-activated serine-threonine kinase), 2jam_B (human, calcium/calmodulin-dependent protein kinase type 1G), and 2y7j_A (human, phosphorylase kinase, Gamma 2) were chosen by I-TASSER as the templates in the modeling. The five most reliable models, defined as model 1, model 2, model 3, model 4 and model 5, respectively, were used for docking. As illustrated in **Fig. 8**, despite moderate sequence identities (around 30% to 37%) between PKD1 and their templates, their 3D structures present similar topologies and overall shapes. Specifically, conserved structure elements of the kinase domain fold into a bi-lobed catalytic core structure, with ATP binding in a deep cleft located between these two lobes. These observations reinforced our strategy to utilize the structural conservation in the PKD1 kinase domain to identify the key residues for inhibitor-protein interactions.

### Molecular docking of the PKD1 selective inhibitors

**Figure 9:**
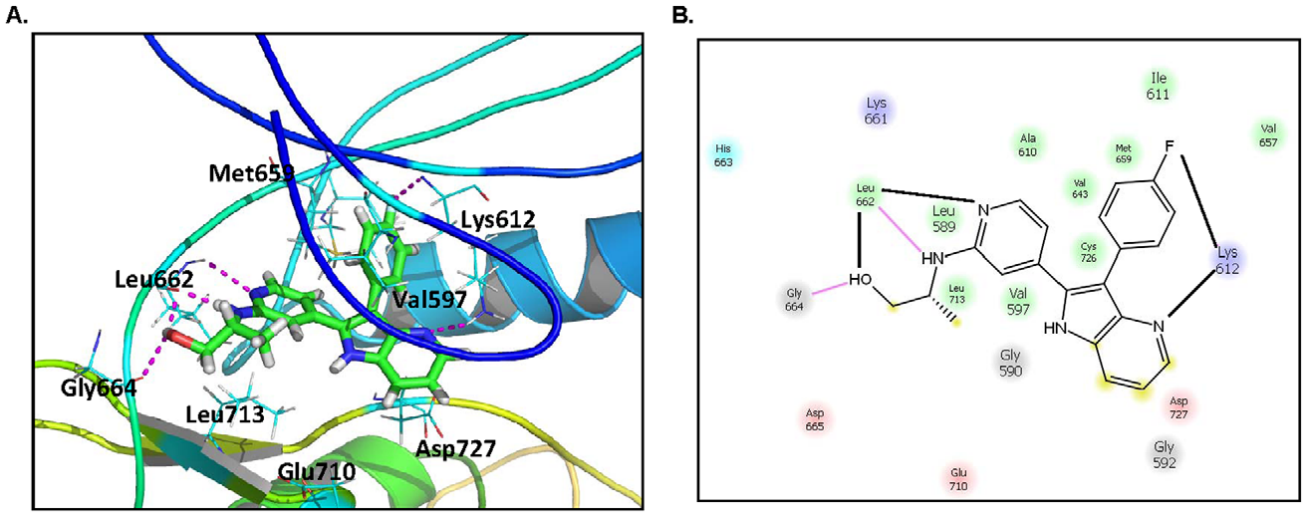
Molecular modeling of compound 139 in the active site of a PKD1 homology model. **A**. The docking result of the bioactive compound **139** in the ATP binding site of the PKD1 kinase domain. *carton ribbon and thick line*, PKD1; *ball and stick*, Compound **139**; *thin line*, residues in the binding pocket; *magenta line*, hydrogen bond. **B**. The proposed key contacts in the active site. *purple line*, hydrogen bond; residues in different colors: *purple*, basic; *pink*, acidic; *green*, hydrophobic; *gray*, hydrophilic.

As a part of the validation process, the quality of models was assessed by molecular docking experiments using Sybyl ×1.3. A total of twenty-eight bioactive PKD1 inhibitors were docked into the ATP binding site of the PKD kinase domain for all five models. The docking results from models 3 and 4, which had the highest docking scores, are shown in **Table S2**. All docking scores ranged from 4 to 9, which can be correlated to K_d_ values of 100 µM to 1 nM, respectively [36]. The docking results were manually checked to make sure that the binding mode of each compound had reasonable interactions and geometry fitting. **Fig. 9** illustrates the predicted interactions between the representative 4-azaindole **139** and the kinase domain of PKD1. The pyridine ring, together with the side chain of **139**, forms hydrogen bonding interactions with the backbone of Leu662 and Gly664, which are located in the hinge region. The fluorine atom on the benzene ring interacts with the backbone -NH of Lys612. Additionally, a hydrogen bond was observed between the nitrogen atom of the pyridine in the 1*H*-pyrrolo[3,2-b]pyridine and the charged side chain of Lys612. These interactions are congruent with the predicted binding mode of experimentally tested previous PKD1 inhibitors [21]. Additional possible docking poses were available, though the interactions at the hinge region in these poses were not consistent with the published docking poses of similar inhibitors in the active site of p38 [32] or those of known PKD1 inhibitors in a homology model [20], [21], i.e. they were considered less favorable (**Fig. S1**). Data from our homology modeling revealed an unoccupied pocket next to the indole nitrogen atom, suggesting that substitution at this position may be well tolerated. However, our data showed a clear preference of non-substituted or small alkyl substituents (compounds **116**, **119**, **121**, **122**, **123**) at this position (Fig. 3). In contrast, substitution of the pyridyl ring at the *ortho*-position to the nitrogen (compounds **139** and **140**) led to significantly enhanced inhibitory activity (from ∼50% inhibition up to ∼90%). This enhanced activity may be due to an additional hydrogen bonding interaction between the monohydroxyamines substituents and Leu662 and Gly664 at the hinge region (**Fig. 9**).

## Discussion

In this study, a small focused library of kinase inhibitors was screened against PKD1 and revealed new scaffolds for selective disruption of ATP binding to this serine/threonine kinase. Starting with 235 compounds, twenty-eight potent inhibitors were further investigated for selectivity against the closely related PKCs and CAMKs, resulting in six highly selective PKD1 inhibitors. Two new scaffold types were represented in the final lead structures (4-azaindoles and a quinolinylmethylenethiazolinone), and a representative member of each type was further evaluated in secondary assays. The concentration dependence *in vitro* and *ex vivo* as well as the kinetic profile was determined. The selectivity of 4-azaindoles was evaluated by a kinetic profile of 353 diverse protein kinases in the human kinome. In summary, our study has identified a novel class of 4-azaindole derivatives as potent PKD inhibitors with selectivity against PKCs and CAMKs. As exemplified by compound **139**, this class of inhibitors inhibited PKD1 in the low nM range, was cell-active, and competitive with ATP for enzyme inhibition.

The PKD inhibitory activity has not been previously demonstrated for the two new scaffolds, thus these are considered novel findings. On the basis of their selectivity for PKCs and CAMKs, we chose to primarily focus on the 4-azaindole series of inhibitors, since they clearly displayed greater selectivity for PKD1 than the quinolinylmethylenethiazolinone derivative. 4-Azaindoles have previously been developed and characterized as inhibitors of p38α/β by Trejo et al. [32]. The *in vitro* IC_50_s for p38α of the six 4-azaindole derivatives are reported as: **116**, 6.5 nM; **121**, 98 nM; **122**, 53 nM; **123**, 172 nM; **139**, 3 nM; **140**, 1 nM. Interestingly, the activities of these analogs for p38 correlate well with their respective activities for PKD1, indicating similar SAR or active site interactions of these compounds with PKD1 and p38α. In the paper by Trejo et al., selectivity profile of one of the two isomers of compound **140** (**42b**) was evaluated against eleven related kinases. The data reported are in general consistent with those in our paper, with the exception of JNK-1 which was found to be inhibited with an IC_50_ of >10 µM, while in our paper JNK-1 was inhibited maximally 100% at 10 µM [32]. This discrepancy could be due to differences in assay format and sensitivity of each detection method. Clearly, more studies are required to further validate the additional targets of compound **140** identified through kinase profiling analysis. With regard to *in vivo* efficacy, as reported by Trejo et al., the 4-azaindole derivatives exhibit rather high metabolic clearance rates and are subjected to oxidative metabolism at three sites, the 4-pyridyl nitrogen, the 4-azaindole nitrogen, and the hydroxylation of the 6-position of the azaindole ring. Compounds **140** and **139** have been designed to reduce the oxidation at the 4-pyridyl nitrogen, a predominant site of metabolic oxidation. As a result of this modification, *in vivo* efficacy has been demonstrated for compounds **140** and **139** in an acute rat model of LPS-stimulated TNFα synthesis, providing favorable pharmacokinetic (PK) parameters for compound **140**. Based on the desirable drug-like physical properties and promising PK/PD values, compound **140**, and most likely **139**, were deemed potent and selective orally available p38 inhibitors [32]. These findings provide strong support for further development of the **140** and **139** series of analogs as drug/lead structures towards potent and selective PKD1 inhibitors, or dual PKD1/p38 inhibitors, with *in vivo* activity. Although a kinase profile reveals a few additional targets of 4-azaindoles, compound **140** in general displayed excellent selectivity as compared to the non-substituted 4,7-azaindoles (i.e. compound **122**), indicating there remains a distinct possibility to achieve greater selectivity through further medicinal chemistry modifications. On the other hand, although it is desirable to obtain sole selectivity for a single kinase, multi-targeted protein kinase inhibitors tailored towards a small subset of kinases with distinct biological functions could be more attractive therapeutically; and, in fact, this strategy has proven to be an effective treatment in oncology. In this regard, PKD inhibitors with dual action on p38α might be equally attractive therapeutically, since both kinases have been implicated in inflammatory responses [37], [38], [39], [40], [41] and cancer development [4], [42].

To further explore the mechanism of actions of these active PKD1 compounds, molecular modeling technologies were utilized to investigate putative binding modes [34], [35]. The three-dimensional structure of PKD1 was built based on high-resolution crystal structures of homologues, and the catalytic (kinase) domain, which consists of two lobes and an intervening linker, was well modeled. Subsequently, docking simulations were carried out, in which all ligands were docked into the putative ATP binding pocket of the kinase domain, and the resulting docking scores were relatively high. The interactions between the active lead compound **139** and the PKD1 kinase domain were further illustrated in detail. The modeling results are congruent with our experimental findings, demonstrating that these compounds are PKD1 inhibitors binding to the ATP site of kinase domain. The computational analyses provide additional insights into the possible molecular interactions and important binding residues of PKD1 and will prove useful in our future pharmacophore refinements.

## Materials and Methods

### Chemicals and Reagents

Kinase active recombinant GST-tagged human protein kinase D1 (PKD1) was obtained from Enzo Life sciences (Farmingdale, NY). DMSO was purchased from Sigma. Recombinant PKCα, PKCδ and CAMKIIα were obtained from SignalChem (Richmond, BC, Canada). ATP was purchased from Fisher Scientific. HDAC5 substrate peptide was synthesized by Biobasic Canada Inc. (Markham, ON). Myelin basic protein 4–14 was purchased from AnaSpec Inc. (Fremont, CA). The targeted kinase inhibitor library was obtained from Hoffmann-La Roche, Inc.

### In vitro radiometric PKD1 screening assay

An *in vitro* radiometric kinase assay was used to screen 235 compound library for PKD1 inhibitory activity at 1 µM concentration. 1.2 µg of HDAC-5 was used as substrate in the reaction. Phosphorylation of HDAC5 was detected in a kinase reaction having 1 µCi [γ-^32^P] ATP (Perkin Elmer Life Sciences), 25 µM ATP, 50 ng purified recombinant PKD1 in 50 µL kinase buffer containing 50 mM Tris-HCl, pH 7.5, 4 mM MgCl_2_ and 10 mM β-mercaptoethanol. The reaction was incubated at 30° for 10 minutes and 25 µL of the reaction was spotted on Whatman P81 filter paper. The filter paper was washed 3 times in 0.5% phosphoric acid, air dried and counted using Beckman LS6500 multipurpose scintillation counter. Percent PKD1 inhibition was graphed using GraphPad Prism software 5.0.

### In vitro radiometric PKC and CAMKIIα kinase assay

The PKC kinase assay was carried out by co-incubating 1 µCi [γ-^32^P]ATP, 20 µM ATP, 50 ng of purified PKCα or PKCδ and 5 µg of myelin basic protein 4–14, 0.25 mg/mL bovine serum albumin, 0.1 mg/mL phosphatidylcholine/phosphatidylserine (80/20%) (1 µM), 1 µM phorbol dibutyrate in 50 µL of kinase buffer containing 50 mM Tris-HCl, pH 7.5, 4 mM MgCl_2_ and 10 mM β-mercaptoethanol. For the CAMK assay, 50 ng of CAMK and 2 µg Syntide-2 substrate in 50 µL kinase buffer were incubated with 0.1 mM MgCl2, 1 µCi of [γ-^32^P] ATP, 70 µM ATP. 0.5 mM CaCl_2_ and 30 ng/µL calmodulin were preincubated for 15 min on ice and then added in the kinase reaction. The reactions were incubated at 30°C for 10 minutes and 25 µL of the reaction was spotted on Whatman P81 filter paper. The filter paper was washed 3 times in 0.5% phosphoric acid, air dried and counted using Beckman LS6500 multipurpose scintillation counter.

### In vitro radiometric p38 kinase assay

Twenty-eight compounds that were found to inhibit PKD1 were screened for p38 inhibitory activity at 1 µM concentration in an *in vitro* radiometric kinase assay. Phosphorylation of 3.5 µg of p38 substrate was detected in a reaction having 1 µCi [γ-^32^P] ATP (Perkin Elmer Life Sciences), 50 µM ATP, 50 ng active recombinant p38 in 50 µL kinase buffer containing 25 mM Tris-HCl, pH 7.5, 10 mM MgCl_2_, 5 mM β-glycerophosphate, 0.1 mM Na_3_VO_4_ and 2 mM DTT. The reaction was incubated at 30°C for 10 minutes and 25 µL of the reaction was spotted on Whatman P81 filter paper. The filter paper was washed 3 times in 0.5% phosphoric acid, air dried and counted using Beckman LS6500 multipurpose scintillation counter. Percent p38 inhibition was graphed using GraphPad Prism software 5.0.

### Homology modeling

The 3D structural model of the kinase domain (residues 587 to 835) of human PKD1 was generated using the I-TASSER server. I-TASSER was ranked as the number 1 server in the recent Critical Assessment of Techniques for Protein Structure Prediction (CASP9, 2010) competition for homology modeling and threading. The I-TASSER combines the methods of threading, ab initio modeling, and structural refinement to build reliable models [43], [44].

### Settings of docking program

All the docking calculations were performed by using Surflex-Dock [45] module of Sybyl ×1.3. The models of PKD1 kinase domain from I-TASSER were first modified by adding all hydrogen atoms. The docking area was defined by a pocket covering the ATP binding site of the protein, including residues Ala610, Lys612, Met659, Glu660, Lys661, Leu662, His663, Glu710, Leu713, and Cys726. These residues were proposed by a reported literature [21]. A protomol (pseudo-binding site) were generated according to these reference residues. Twenty additional starting conformations per molecule were configured in the docking process to search for a good docking mode. We treated all the models with the same protocol and docking was run with default settings for all other parameters.

### Structural comparison between our compounds and known PKD1 inhibitors

To evaluate the similarity between our active compounds and those known PKD1 inhibitors, both 2D and 3D similarity methodologies were employed to compare these 8 compounds against 12 previously reported PKD1 inhibitors [27] and ATP. The Surflex-Sim 3D similarity program was used for the calculation of morphological similarity score (MSS, scores range from 0.0 to 10.0, where large value means two compounds are very similar in 3D shapes) [46] and UNITY 2D search was used for the calculation of the Tanimoto score (TS, scores range from 0.0 to 1.0, where large TS score implies two chemicals are 2D structural resemble as well).

### Kinase profiling

The global kinase profiling experiment was performed by Ambit Biosciences [47].

## Supporting Information

Figure S1 **Three alternative docking poses of compound 139 in the PKD1 kinase domain.** *carton ribbon and thick line*, PKD1; *ball and stick*, Compound **139**; *thin line*, residues in the binding pocket; *magenta line*, hydrogen bond.
(DOCX)

Table S1 **Structural dissimilarity of PKD1 inhibitors and known PKD1 inhibitors.** MSS, Morphological Similarity Score; TS, Tanimoto Score. 13c: a 1-naphthyridine analog; 24c: a 3, 5-diarylazole analog.
(DOCX)

Table S2 **Docking results of 28 PKD1 inhibitors.** The six lead compounds are bolded.
(DOCX)

File S1 **Kinase profiling of compound 122 and 140.** Kinomescan of compound **122** and **140** was conducted on 353 protein kinases at 10 µM.
(XLS)

## References

[1] Manning G, Whyte DB, Martinez R, Hunter T, Sudarsanam S (2002) The protein kinase complement of the human genome. Science 298: 1912–1934. 12471243 10.1126/science.1075762

[2] Wang QJ (2006) PKD at the crossroads of DAG and PKC signaling. Trends Pharmacol Sci 27: 317–323. 16678913 10.1016/j.tips.2006.04.003

[3] Rozengurt E (2011) Protein kinase D signaling: multiple biological functions in health and disease. Physiology (Bethesda) 26: 23–33. 21357900 10.1152/physiol.00037.2010 PMC4381749

[4] LaValle CR, George KM, Sharlow ER, Lazo JS, Wipf P, et al (2010) Protein kinase D as a potential new target for cancer therapy. Biochim Biophys Acta 1806: 183–192. 20580776 10.1016/j.bbcan.2010.05.003 PMC2947595

[5] Ochi N, Tanasanvimon S, Matsuo Y, Tong Z, Sung B, et al (2011) Protein kinase D1 promotes anchorage-independent growth, invasion, and angiogenesis by human pancreatic cancer cells. J Cell Physiol 226: 1074–1081. 20857418 10.1002/jcp.22421

[6] Rennecke J, Rehberger PA, Furstenberger G, Johannes FJ, Stohr M, et al (1999) Protein-kinase-Cmu expression correlates with enhanced keratinocyte proliferation in normal and neoplastic mouse epidermis and in cell culture. Int J Cancer 80: 98–103. 9935238 10.1002/(sici)1097-0215(19990105)80:1<98::aid-ijc19>3.0.co;2-d

[7] Ristich VL, Bowman PH, Dodd ME, Bollag WB (2006) Protein kinase D distribution in normal human epidermis, basal cell carcinoma and psoriasis. Br J Dermatol 154: 586–593. 16536798 10.1111/j.1365-2133.2005.07073.x

[8] Biswas MH, Du C, Zhang C, Straubhaar J, Languino LR, et al (2010) Protein kinase D1 inhibits cell proliferation through matrix metalloproteinase-2 and matrix metalloproteinase-9 secretion in prostate cancer. Cancer Res 70: 2095–2104. 20160036 10.1158/0008-5472.CAN-09-4155 PMC3197700

[9] Chen J, Deng F, Singh SV, Wang QJ (2008) Protein kinase D3 (PKD3) contributes to prostate cancer cell growth and survival through a PKCepsilon/PKD3 pathway downstream of Akt and ERK 1/2. Cancer Res 68: 3844–3853. 18483269 10.1158/0008-5472.CAN-07-5156

[10] Eiseler T, Doppler H, Yan IK, Goodison S, Storz P (2009) Protein kinase D1 regulates matrix metalloproteinase expression and inhibits breast cancer cell invasion. Breast Cancer Res 11: R13. 19243594 10.1186/bcr2232 PMC2687718

[11] Kim M, Jang HR, Kim JH, Noh SM, Song KS, et al (2008) Epigenetic inactivation of protein kinase D1 in gastric cancer and its role in gastric cancer cell migration and invasion. Carcinogenesis 29: 629–637. 18283041 10.1093/carcin/bgm291

[12] Azoitei N, Kleger A, Schoo N, Thal DR, Brunner C, et al (2011) Protein kinase D2 is a novel regulator of glioblastoma growth and tumor formation. Neuro Oncol 13: 710–724. 21727210 10.1093/neuonc/nor084 PMC3129279

[13] Fielitz J, Kim MS, Shelton JM, Qi X, Hill JA, et al (2008) Requirement of protein kinase D1 for pathological cardiac remodeling. Proc Natl Acad Sci U S A 105: 3059–3063. 18287012 10.1073/pnas.0712265105 PMC2268584

[14] Harrison BC, Kim MS, van Rooij E, Plato CF, Papst PJ, et al (2006) Regulation of cardiac stress signaling by protein kinase d1. Mol Cell Biol 26: 3875–3888. 16648482 10.1128/MCB.26.10.3875-3888.2006 PMC1488991

[15] Vega RB, Harrison BC, Meadows E, Roberts CR, Papst PJ, et al (2004) Protein kinases C and D mediate agonist-dependent cardiac hypertrophy through nuclear export of histone deacetylase 5. Mol Cell Biol 24: 8374–8385. 15367659 10.1128/MCB.24.19.8374-8385.2004 PMC516754

[16] Bossuyt J, Helmstadter K, Wu X, Clements-Jewery H, Haworth RS, et al (2008) Ca2+/Calmodulin-Dependent Protein Kinase II{delta} and Protein Kinase D Overexpression Reinforce the Histone Deacetylase 5 Redistribution in Heart Failure. Circ Res 10.1161/CIRCRESAHA.107.169755 18218981

[17] Sharlow ER, Giridhar KV, Lavalle CR, Chen J, Leimgruber S, et al (2008) Potent and selective disruption of protein kinase d functionality by a benzoxoloazepinolone. J Biol Chem 283: 33516–33526. 18829454 10.1074/jbc.M805358200 PMC2586241

[18] George KM, Frantz MC, Bravo-Altamirano K, Lavalle CR, Tandon M, et al (2011) Design, Synthesis, and Biological Evaluation of PKD Inhibitors. Pharmaceutics 3: 186–228. 22267986 10.3390/pharmaceutics3020186 PMC3261798

[19] Monovich L, Vega RB, Meredith E, Miranda K, Rao C, et al (2010) A novel kinase inhibitor establishes a predominant role for protein kinase D as a cardiac class IIa histone deacetylase kinase. FEBS Lett 584: 631–637. 20018189 10.1016/j.febslet.2009.12.014

[20] Meredith EL, Beattie K, Burgis R, Capparelli M, Chapo J, et al (2010) Identification of potent and selective amidobipyridyl inhibitors of protein kinase D. J Med Chem 53: 5422–5438. 20684592 10.1021/jm100076w

[21] Meredith EL, Ardayfio O, Beattie K, Dobler MR, Enyedy I, et al (2010) Identification of orally available naphthyridine protein kinase D inhibitors. J Med Chem 53: 5400–5421. 20684591 10.1021/jm100075z

[22] Gamber GG, Meredith E, Zhu Q, Yan W, Rao C, et al (2011) 3,5-diarylazoles as novel and selective inhibitors of protein kinase D. Bioorg Med Chem Lett 21: 1447–1451. 21300545 10.1016/j.bmcl.2011.01.014

[23] Harikumar KB, Kunnumakkara AB, Ochi N, Tong Z, Deorukhkar A, et al (2010) A novel small-molecule inhibitor of protein kinase D blocks pancreatic cancer growth in vitro and in vivo. Mol Cancer Ther 9: 1136–1146. 20442301 10.1158/1535-7163.MCT-09-1145 PMC2905628

[24] Evans IM, Bagherzadeh A, Charles M, Raynham T, Ireson C, et al (2010) Characterization of the biological effects of a novel protein kinase D inhibitor in endothelial cells. Biochem J 429: 565–572. 20497126 10.1042/BJ20100578 PMC2907712

[25] Bravo-Altamirano K, George KM, Frantz MC, Lavalle CR, Tandon M, et al (2011) Synthesis and Structure-Activity Relationships of Benzothienothiazepinone Inhibitors of Protein Kinase D. ACS Med Chem Lett 2: 154–159. 21617763 10.1021/ml100230n PMC3100199

[26] Lavalle CR, Bravo-Altamirano K, Giridhar KV, Chen J, Sharlow E, et al (2010) Novel protein kinase D inhibitors cause potent arrest in prostate cancer cell growth and motility. BMC Chem Biol 10: 5. 20444281 10.1186/1472-6769-10-5 PMC2873968

[27] Sharlow ER, Mustata Wilson G, Close D, Leimgruber S, Tandon M, et al (2011) Discovery of diverse small molecule chemotypes with cell-based PKD1 inhibitory activity. PLoS One 6: e25134. 21998636 10.1371/journal.pone.0025134 PMC3187749

[28] Zugaza JL, Sinnett-Smith J, Van Lint J, Rozengurt E (1996) Protein kinase D (PKD) activation in intact cells through a protein kinase C-dependent signal transduction pathway. Embo J 15: 6220–6230. 8947045 PMC452445

[29] Rozengurt E, Rey O, Waldron RT (2005) Protein kinase D signaling. J Biol Chem 280: 13205–13208. 15701647 10.1074/jbc.R500002200

[30] Waldron RT, Rey O, Iglesias T, Tugal T, Cantrell D, et al (2001) Activation loop Ser744 and Ser748 in protein kinase D are transphosphorylated in vivo. J Biol Chem 276: 32606–32615. 11410586 10.1074/jbc.M101648200

[31] Matthews SA, Rozengurt E, Cantrell D (1999) Characterization of serine 916 as an in vivo autophosphorylation site for protein kinase D/Protein kinase Cmu. J Biol Chem 274: 26543–26549. 10473617 10.1074/jbc.274.37.26543

[32] Trejo A, Arzeno H, Browner M, Chanda S, Cheng S, et al (2003) Design and synthesis of 4-azaindoles as inhibitors of p38 MAP kinase. J Med Chem 46: 4702–4713. 14561090 10.1021/jm0301787

[33] Chen S, Chen L, Le NT, Zhao C, Sidduri A, et al (2007) Synthesis and activity of quinolinyl-methylene-thiazolinones as potent and selective cyclin-dependent kinase 1 inhibitors. Bioorg Med Chem Lett 17: 2134–2138. 17303421 10.1016/j.bmcl.2007.01.081

[34] Chen JZ, Wang J, Xie XQ (2007) GPCR structure-based virtual screening approach for CB2 antagonist search. J Chem Inf Model 47: 1626–1637. 17580929 10.1021/ci7000814

[35] Xie XQ, Chen JZ, Billings EM (2003) 3D structural model of the G-protein-coupled cannabinoid CB2 receptor. Proteins: Structure, Function, and Bioinformatics 53: 307–319. 10.1002/prot.10511 14517981

[36] Meurice N, Wang L, Lipinski CA, Yang Z, Hulme C, et al (2010) Structural conservation in band 4.1, ezrin, radixin, moesin (FERM) domains as a guide to identify inhibitors of the proline-rich tyrosine kinase 2. J Med Chem 53: 669–677. 20017492 10.1021/jm901247a PMC3178892

[37] Kim YI, Park JE, Brand DD, Fitzpatrick EA, Yi AK (2010) Protein kinase D1 is essential for the proinflammatory response induced by hypersensitivity pneumonitis-causing thermophilic actinomycetes Saccharopolyspora rectivirgula. J Immunol 184: 3145–3156. 20142359 10.4049/jimmunol.0903718 PMC2987577

[38] Yamashita K, Gon Y, Shimokawa T, Nunomura S, Endo D, et al (2010) High affinity receptor for IgE stimulation activates protein kinase D augmenting activator protein-1 activity for cytokine producing in mast cells. Int Immunopharmacol 10: 277–283. 19932769 10.1016/j.intimp.2009.11.011

[39] Tan M, Hao F, Xu X, Chisolm GM, Cui MZ (2009) Lysophosphatidylcholine activates a novel PKD2-mediated signaling pathway that controls monocyte migration. Arterioscler Thromb Vasc Biol 29: 1376–1382. 19520973 10.1161/ATVBAHA.109.191585 PMC3073140

[40] Park JE, Kim YI, Yi AK (2009) Protein kinase D1 is essential for MyD88-dependent TLR signaling pathway. J Immunol 182: 6316–6327. 19414785 10.4049/jimmunol.0804239 PMC2683622

[41] Ivison SM, Graham NR, Bernales CQ, Kifayet A, Ng N, et al (2007) Protein kinase D interaction with TLR5 is required for inflammatory signaling in response to bacterial flagellin. J Immunol 178: 5735–5743. 17442957 10.4049/jimmunol.178.9.5735

[42] Sundram V, Chauhan SC, Jaggi M (2011) Emerging Roles of Protein Kinase D1 in Cancer. Mol Cancer Res 10.1158/1541-7786.MCR-10-0365 PMC4030726 21680539

[43] Zhu H, Yang Y, Zhang H, Han Y, Li Y, et al (2008) Interaction between protein kinase D1 and transient receptor potential V1 in primary sensory neurons is involved in heat hypersensitivity. Pain 137: 574–588. 18063480 10.1016/j.pain.2007.10.025

[44] Zhang Y (2008) I-TASSER server for protein 3D structure prediction. BMC Bioinformatics 9: 40. 18215316 10.1186/1471-2105-9-40 PMC2245901

[45] Jain AN (2003) Surflex: fully automatic flexible molecular docking using a molecular similarity-based search engine. J Med Chem 46: 499–511. 12570372 10.1021/jm020406h

[46] Yera ER, Cleves AE, Jain AN (2011) Chemical structural novelty: on-targets and off-targets. J Med Chem 54: 6771–6785. 21916467 10.1021/jm200666a PMC3188662

[47] Fabian MA, Biggs WH 3rd, Treiber DK, Atteridge CE, Azimioara MD, et al (2005) A small molecule-kinase interaction map for clinical kinase inhibitors. Nat Biotechnol 23: 329–336. 15711537 10.1038/nbt1068

