# Supplementary material for: A Targeted Library Screen Reveals a New Inhibitor Scaffold for Protein Kinase D
Source: PLoS One. 2012 Sep 18;7(9):e44653. doi: 10.1371/journal.pone.0044653 (PMC3445516; doi:10.1371/journal.pone.0044653)
Supplement: Figure S1 — Three alternative docking poses of compound 139 in the PKD1 kinase domain. carton ribbon and thick line, PKD1; ball and stick, Compound 139; thin line, residues in the binding pocket; magenta line, hydrogen bond. (DOCX) [file pone.0044653.s001.docx]

**Figure S1. Three alternative docking poses of compound 139 in the PKD1 kinase domain.** *carton ribbon and thick line*, PKD1; *ball and stick*, Compound 139; *thin line*, residues in the binding pocket; *magenta line*, hydrogen bond.
