# Supplementary material for: A Targeted Library Screen Reveals a New Inhibitor Scaffold for Protein Kinase D
Source: PLoS One. 2012 Sep 18;7(9):e44653. doi: 10.1371/journal.pone.0044653 (PMC3445516; doi:10.1371/journal.pone.0044653)
Supplement: Table S1 — Structural dissimilarity of PKD1 inhibitors and known PKD1 inhibitors. MSS, Morphological Similarity Score; TS, Tanimoto Score. 13c: a 1-naphthyridine analog; 24c: a 3, 5-diarylazole analog. (DOCX) [file pone.0044653.s002.docx]

**Table S1. Structural dissimilarity of PKD1 inhibitors and known PKD1 inhibitors.** MSS, Morphological Similarity Score; TS, Tanimoto Score. 13c: a 1-naphthyridine analog; 24c: a 3, 5-diarylazole analog.

| **Similarity**  **scores** | **121**  UPCMLDRO1155240000 | | **122**  UPCMLDRO1155697000 | | **123**  UPCMLDRO1155798000 | | **139**  UPCMLDRO3202312001 | | **140** UPCMLDRO3206145001 | | **209**  UPCMLDRO4595949000 | |
| --- | --- | --- | --- | --- | --- | --- | --- | --- | --- | --- | --- | --- |
|  | **MSS** | **TS** | **MSS** | **TS** | **MSS** | **TS** | **MSS** | **TS** | **MSS** | **TS** | **MSS** | **TS** |
| **ATP** | 5.26 | 0.272 | 5.85 | 0.265 | 6.05 | 0.322 | 5.47 | 0.290 | 5.76 | 0.305 | 6.06 | 0.215 |
| **CID755673** | 5.69 | 0.225 | 5.57 | 0.201 | 6.49 | 0.243 | 6.96 | 0.223 | 7.08 | 0.228 | 6.80 | 0.230 |
| **kb-NB142-70** | 4.47 | 0.204 | 4.83 | 0.177 | 5.64 | 0.218 | 5.46 | 0.207 | 5.46 | 0.212 | 5.81 | 0.212 |
| **CID1893668** | 4.78 | 0.158 | 3.29 | 0.174 | 4.12 | 0.174 | 4.39 | 0.234 | 4.61 | 0.235 | 4.21 | 0.204 |
| **CID2011756** | 4.24 | 0.248 | 3.46 | 0.219 | 3.85 | 0.272 | 4.23 | 0.289 | 4.38 | 0.291 | 4.33 | 0.225 |
| **CID5389142** | 4.84 | 0.326 | 4.60 | 0.354 | 5.28 | 0.332 | 6.80 | 0.434 | 7.10 | 0.456 | 5.26 | 0.236 |
| **BPKDi** | 5.54 | 0.368 | 5.77 | 0.362 | 5.87 | 0.368 | 5.67 | 0.513 | 5.49 | 0.523 | 5.51 | 0.266 |
| **13c** | 6.01 | 0.375 | 5.61 | 0.379 | 6.35 | 0.369 | 5.97 | 0.544 | 5.51 | 0.530 | 6.21 | 0.282 |
| **24c** | 5.09 | 0.309 | 4.91 | 0.273 | 4.83 | 0.324 | 5.94 | 0.334 | 5.91 | 0.340 | 5.33 | 0.241 |
| **CRT5** | 5.63 | 0.316 | 5.99 | 0.342 | 5.32 | 0.404 | 5.73 | 0.410 | 5.76 | 0.282 | 5.63 | 0.316 |
