# Supplementary material for: A Targeted Library Screen Reveals a New Inhibitor Scaffold for Protein Kinase D
Source: PLoS One. 2012 Sep 18;7(9):e44653. doi: 10.1371/journal.pone.0044653 (PMC3445516; doi:10.1371/journal.pone.0044653)
Supplement: Table S2 — Docking results of 28 PKD1 inhibitors. The six lead compounds are bolded. (DOCX) [file pone.0044653.s003.docx]

Table S2. Docking results of 28 PKD1 inhibitors. The six lead compounds are bolded.

| Numerical ID | UPCMLD ID | Inhibition | Model3 | | | Model4 | | |
| --- | --- | --- | --- | --- | --- | --- | --- | --- |
|  |  |  | Total score | Crash | Polar | Total score | Crash | Polar |
| 4 | UPCMLDRO0272148000 | 57% | 5.44 | -0.447 | 3.23 | 4.93 | -0.421 | 0.811 |
| 5 | UPCMLDRO0272159000 | 55% | 4.89 | -0.672 | 0.573 | 5.96 | -0.913 | 3.09 |
| 11 | UPCMLDRO0281601001 | 94% | 6.90 | -1.37 | 2.00 | 7.27 | -1.24 | 3.81 |
| 13 | UPCMLDRO0282155000 | 80% | 8.15 | -1.28 | 2.10 | 7.26 | -1.98 | 2.72 |
| 15 | UPCMLDRO0282986001 | 97% | 7.81 | -1.30 | 1.78 | 6.76 | -0.837 | 1.49 |
| 16 | UPCMLDRO0283049001 | 95% | 7.89 | -1.91 | 5.59 | 7.61 | -0.867 | 3.07 |
| 17 | UPCMLDRO0283120000 | 97% | 7.52 | -2.70 | 3.30 | 8.03 | -3.49 | 4.92 |
| 25 | UPCMLDRO0317253000 | 54% | 5.54 | -0.29 | 1.26 | 5.55 | -1.96 | 2.22 |
| 30 | UPCMLDRO0317340000 | 65% | 6.55 | -1.43 | 0.510 | 5.74 | -3.70 | 2.18 |
| 34 | UPCMLDRO0317377000 | 65% | 6.89 | -1.55 | 2.73 | 7.60 | -0.939 | 1.79 |
| 100 | UPCMLDRO0480500002 | 77% | 8.33 | -3.97 | 2.10 | 8.14 | -3.52 | 4.80 |
| 101 | UPCMLDRO0504833000 | 86% | 6.36 | -0.634 | 3.43 | 6.80 | -0.623 | 3.26 |
| 102 | UPCMLDRO0504985000 | 93% | 7.62 | -1.75 | 5.47 | 6.72 | -0.630 | 5.98 |
| 104 | UPCMLDRO0506220000 | 65% | 6.53 | -2.70 | 2.44 | 7.86 | -1.03 | 2.47 |
| 116 | UPCMLDRO1153853000 | 86% | 5.28 | -0.969 | 1.93 | 4.98 | -0.904 | 1.09 |
| **121** | **UPCMLDRO1155240000** | **55%** | **5.38** | **-0.483** | **0.270** | **4.79** | **-0.291** | **0.0153** |
| **122** | **UPCMLDRO1155697000** | **62%** | **5.01** | **-0.880** | **0.825** | **5.40** | **-1.25** | **2.39** |
| **123** | **UPCMLDRO1155798000** | **58%** | **7.34** | **-0.858** | **2.21** | **6.60** | **-2.75** | **3.13** |
| **139** | **UPCMLDRO3202312001** | **94%** | **9.02** | **-0.829** | **2.27** | **8.32** | **-1.43** | **3.75** |
| **140** | **UPCMLDRO3206145001** | **80%** | **7.71** | **-1.77** | **4.19** | **8.40** | **-1.62** | **2.06** |
| 172 | UPCMLDRO4241967000 | 62% | 3.51 | -0.383 | 0.90 | 6.01 | -0.931 | 2.71 |
| 175 | UPCMLDRO4367842001 | 78% | 7.59 | -1.20 | 2.33 | 6.75 | -1.33 | 2.30 |
| 178 | UPCMLDRO4442080000 | 55% | 5.96 | -1.35 | 1.02 | 6.29 | 1.03 | 1.55 |
| 190 | UPCMLDRO4503319000 | 49% | 6.84 | -1.39 | 0.0914 | 7.19 | -1.57 | 3.76 |
| 198 | UPCMLDRO4509200000 | 62% | 7.20 | -1.61 | 0.260 | 7.59 | -1.11 | 2.46 |
| 203 | UPCMLDRO4554339000 | 85% | 6.12 | -1.33 | 0.172 | 5.70 | -1.96 | 1.75 |
| 205 | UPCMLDRO4569139000 | 82% | 5.41 | -1.03 | 2.52 | 4.81 | -0.967 | 1.96 |
| **209** | **UPCMLDRO4595949000** | **64%** | **4.92** | **-0.703** | **1.79** | **4.70** | **-1.17** | **2.23** |
